# The impact of nutrition on visual cognitive performance in the nutrition, vision, and cognition in sport study

**DOI:** 10.3389/fnut.2023.1208890

**Published:** 2023-06-23

**Authors:** Karen M. Beathard, Nicos Georghiades, Jenna B. Goulart, Aaron J. Riviere, Caroline Sullivan, Melanie Mascarro, Steven E. Riechman

**Affiliations:** ^1^Department of Nutrition, Texas A&M University, College Station, TX, United States; ^2^Department of Kinesiology and Sport Management, Texas A&M University, College Station, TX, United States

**Keywords:** carbohydrates, protein, micronutrients, lutein, zeaxanthin, cognition, cognitive performance, vision

## Abstract

**Introduction:**

The purpose of this study was to examine the influence of nutritional intake on visual perceptual-cognitive performance (VCP) in young healthy adults.

**Methods:**

Ninety-eight healthy men (*n* = 38) and women (*n* = 60) aged 18–33 years participated and maintained their usual dietary intake throughout the study. VCP was measured using the NeuroTracker^™^ CORE (NT) 3-Dimensional (3-D) software program (15 training sessions) over a 15-day period. Food logs and extensive lifestyle measures including body composition, cardiovascular health, sleep and exercise patterns, and general readiness to perform were collected. Mean intake from 10 food logs collected over the 15 days were analyzed using Nutribase software. Statistical analyses were performed in SPSS using repeated measures ANOVA including significant covariates when appropriate.

**Results:**

Males consumed significantly more calories, macronutrients, cholesterol, choline, and zinc and performed significantly better on VCP than the females. Participants who consumed more than 40% of kcals from carbohydrates (*p* = 0.038), less than 24% of kcals from protein (*p* = 0.009), more than 2,000 μg/day lutein/zeaxanthin or more than 1.8 mg/ day vitamin B2 performed significantly better on VCP than those who consumed less than those amounts, respectively.

**Discussion:**

VCP is an important dimension of cognitive function and in the present study is influenced by higher carbohydrate, lutein/ zeaxanthin, and vitamin B2 dietary intake while high protein consumption and the female sex negatively impacted VCP.

## 1. Introduction

Traditionally, athletes spend a lot of time physically training skeletal muscle and cardiovascular systems to enhance sports performance. However, an increasing prevalence of studies suggest that vision and perceptual-cognitive processing are also critical factors that enhance or limit overall sports performance (1–4). While both visual “hardware” (eye health) and “software” (visual processing) domains have been evaluated in relation to visual cognitive ability, the relative importance of each of these appears dependent on the requirements of the specific sport (3–7). Athletes ranging from hockey players to baseball players have unique visual processing requirements that impact their overall athletic performance (1, 3–7).

Computer technology in sport-specific vision training programs may be used to enhance perceptual cognitive ability and testing has been associated with overall sport success (1, 2, 8–10). The 3-dimensional (3D) multi-object-tracking (MOT) is a robust computerized training intervention that requires tracking multiple objects in 3D that are moving among distractors in a broad field using a speed threshold to measure performance (8–10). This technology sensitively differentiates low and high visual-cognitive performance, requires peripheral vision, individualizes speed and difficulty levels, and consistently challenges trainees to progress (10). The NeuroTracker^™^ CORE (NT) 3D software program from CogniSens Inc. is a single-task integrative perceptual cognitive training system that is equivalent to the 3D-MOT system. It evaluates the individual’s perceptual cognitive ability, provides training, and has been shown to be effective in improving cognitive performance after a specified number of training sessions (8, 9).

Previous investigations using the NT 3D-MOT system utilized maximal speed thresholds and adaptive training responses to differentiate between professional, elite amateurs, and non-athletes. Professional athletes demonstrated significantly greater perceptual cognitive processing with a much steeper learning curve in a dynamic environment when compared to elite amateur athletes. Similarly, elite amateur athletes performed significantly better and had a significantly steeper learning curve than non-athletes (8). College age non-athletes, who completed 10 NT training sessions, experienced significant improvements in cognitive performance, attention span, memory, and reaction time, in addition to quantitative changes in brain function when comparing baseline and final sessions (10).

While the importance of visual processing speed in sport performance is increasingly recognized, the understanding of the variability between individuals is poor. Individual intake of macronutrients including the dietary ratio of carbohydrates, protein, and fat appear to impact overall cognitive ability. Some studies report mixed effects of high carbohydrate diets that are dependent on the type of carbohydrate consumed. Simple carbohydrates have been shown to reduce cognitive function as well as lead to pathological cognitive decline through deleterious effects on the brain. Complex carbohydrates with high fiber content are reported to be beneficial for cognition through improved blood glucose control and thus reduce metabolic dysregulation. Studies also support a balanced protein intake due to evidence that inadequate or overconsumption of protein can negatively impact cognition while adequate intake improves certain cognitive tests. Additionally, research supports higher intakes in healthy fats. For example, polyunsaturated fats are linked to improved cognitive function while diets high in saturated fatty acids and cholesterol are shown to have a negative correlation with global cognitive function (11–18).

Micronutrient intake can also greatly influence nervous and sensory function including choline, B vitamins, calcium, magnesium, zinc, copper, beta-carotene, vitamins C and E, ω-3 fatty acids, lutein/zeaxanthin. These nutrients have been studied most often for their role in visual cognitive function, performance and/or eye health (19–26). Lutein/zeaxanthin also positively influenced visual cognitive performance by serving as antioxidant and anti-inflammatory agents to reduce oxidative stress and inflammation, filtering short-wave blue light that damage the retina and diminish visual sharpness and supporting retinal light processing through synaptic membrane regulation (2, 20, 21, 26). The follow-up to the Age-Related Eye Disease Study 2 (AREDS2) study reported lutein/zeaxanthin contributed to eye health by positively impacting those with late age-related macular degeneration (AMD) (26).

The purpose of the IONSport study was to examine the influence of free-living nutritional intake on visual perceptual-cognitive performance in young healthy adults. The focus was to examine macronutrients and micronutrients in the current literature that reportedly impact eye health and/or cognitive performance. We employed a cross-sectional design to characterize cognitive performance and dietary intake. Participants kept food logs on each day of cognitive testing to evaluate the effect of variability in a free-living diet on cognitive performance; detailed 10-day nutrient averages were used to represent individual long term nutrient intake. The NT 3D-MOT software program was used to identify individual perceptual cognitive ability. We are unaware of any studies that have evaluated the relationship of nutrient intake to visual cognitive testing using the NT software.

## 2. Materials and methods

### 2.1. Subjects

Men and women aged 18–33 years were recruited for the IONSport study using a convenience sampling strategy. Email, posted fliers, and direct contact with student groups on the Texas A&M University campus were used to recruit participants. Individuals with a body mass index (BMI) less than 18, had a pacemaker, experienced vertigo, or had difficulty with 3D viewing were excluded. Persons who self-reported their inability to distinguish between yellow and orange due to colorblindness on a pre-screening form were also excluded since the training program required the ability to distinguish these colors. All participants signed an informed consent, and the Texas A&M University Human Subject’s Institutional Review Board (IRB) approved the study protocol.

### 2.2. Procedures

Prior to the start of the study, baseline data including blood pressure (BP); heart rate (HR) (Omron Healthcare, Inc. Bannockburn, IL, United States); visual acuity using the Snellen Chart; and body composition using the Biometric Beurer BF 520 BIA technology (Beurer, Germany) were collected (27). Subjects completed the Pittsburgh Sleep Quality Index, medical history, and the Modifiable Activity Questionnaire to provide detailed sleep, health, and physical activity background (28, 29). Participants came to the lab 10 times over a 15-day period to complete cognitive testing. Daily data, anthropometrics, and food logs were collected at each visit.

### 2.3. Nutrition education and monitoring

Subjects were instructed to continue their usual eating behaviors and physical activity throughout the study. A registered dietitian nutritionist (RDN) prepared and emailed five, one-to-two-minute, online instructional videos and provided participants direct guidance on how to accurately assess and document food and beverages consumed. Subjects were asked to log all food and beverages consumed on each of the 10 cognitive training days. Each food log was reviewed daily by an RDN and, if necessary, additional details were collected to ensure logs were complete and accurate. NutriBase 19 Pro Edition, v. 19.2 software (NB) (CyberSoft, Inc., Houston, TX) was used for dietary analyses.

### 2.4. Visual cognitive performance testing

Participants completed 15 cognitive training sessions using the NT 3D software program over 10 training days that included alternating single and double training sessions with 4–5 interspersed days of no training (15 total days of the study). During the first training session, the cognitive testing procedures were explained to participants. Participants were seated in a chair aligned 4½’ from a 50” 3D television in a dark, quiet testing room with the seat adjusted so that their eyes were positioned at the center of the screen. They wore active 3D glasses that directly interacted with 3D software and noise-canceling headphones to minimize distractions. Participants who relied on glasses for corrected vision wore them under the 3D glasses when training.

Each training session included tracking the spatial location of four pre-identified target spheres that were initially a distinct color from the other four spheres. Once identified, these spheres became identical in color to the four other spheres. All eight identical spheres moved among each other at a given speed within a 3D virtual space, passing in front of or behind each other, colliding with each other or the edges of the screen and changing directions. After 6 s of movement, the spheres stopped, and the participant picked out the four pre-identified spheres. If the subject selected all four of the correct spheres, the speed of sphere movement increased for the next 6 s trial. If one or more spheres was missed, the speed of sphere movement decreased for the next trial in a 0.05 log up 0.05 log down pattern (30, 31) subjects performed 20 trials within a single training session obtaining a “speed threshold,” the level at which the participant correctly tracked and selected the correct objects 50% of the time. The final speed threshold for each training session and the progression over 15 sessions were the primary determinants of cognitive performance.

Each of the 10 cognitive training days included collection of recent physical activity, fluid intake, most recent urine color (validated urine color scale), blood pressure, heart rate, readiness to perform, body composition, level of sleepiness (Stanford Sleepiness questionnaire) and actual hours of sleep the previous night (32, 33).

### 2.5. Statistical analyses

IBM SPSS 27.0 software (SPSS Inc., Chicago, IL) was used for all the analyses. NB was used to calculate the 10-day food log mean of individual nutrients which was used in cognitive performance analyses. Lutein/zeaxanthin were analyzed together since NB reported these nutrients as one. Visual cognitive performance was assessed using the 15-day NT speed threshold. Speed threshold analysis included: (1) evaluating mean and maximal performance and (2) comparing baseline sessions 1–3 to the final 3 sessions. The relationship of nutritional intake and cognitive performance was evaluated based on sex and comparison of top and poor performers on the NT cognitive tests. Data are presented as estimated mean ± SD, and *p* < 0.05 was considered statistically significant.

A repeated measures analysis of variance (ANOVA) was performed to compare the effect of nutrient intake on visual cognitive performance. Nutrients which showed suggestive positive or negative associations to cognitive performance parameters were further analyzed by categorizing intakes according to recommended levels and natural groupings within the data. Stepwise linear regression was used to determine the independent effect of cognitive performance using variables that continued to show a consistent effect on cognitive performance parameters.

## 3. Results

### 3.1. Subjects

The IONSport Study enrolled 109 participants, and 98 (38 males, 60 females) had complete data sets for analyses (at least 9 daily food records, 15 NT sessions, and at least 9 site visits in 15 days). Table 1 shows the characteristics of all male and female participants. There were significant differences among male and female participants in age, height, weight, lean mass, BMI, and percent body fat. Regarding blood pressure, there was a significant difference in the systolic but not diastolic blood pressure. There was also a significant difference in hydration status as indicated by urine color. There was no significance between the hours of sleep or responses on the Stanford Sleepiness scale. Most participants were Caucasian (49%).

**Table 1:** Participant characteristics.

	Male (*n* = 38)		Female (*n* = 60)		
	Mean	SD	Mean	SD	P (sex)
Age (years)	22.7	3.0	21.1	3.6	0.032
Height (m)	1.78	0.09	1.64	0.06	<0.001
Weight (kg)	80.5	10.4	60.9	9.3	<0.001
Lean mass (kg)	69.8	7.9	46.0	4.8	<0.001
BMI (kg•m^−2^)	25.3	2.7	22.5	3.2	<0.001
Systolic BP (mmHg)	122.8	10.4	108.4	8.9	<0.001
Diastolic BP (mmHg)	73.4	4.9	71.3	7.4	0.159
Fat (%)	12.9	3.9	23.8	5.5	<0.001
Urine color (AU)	2.6	0.9	2.1	0.6	0.002
Sleep (hours)	6.8	0.9	6.8	0.9	0.871
Stanford sleepiness	2.3	0.9	2.3	0.6	0.859
Race (% white)	40.5		55.1		

### 3.2. Average dietary intake and cognitive performance

There was variance between men and women in both average dietary intake and cognitive performance outcomes as displayed in Table 2. Males consumed significantly more calories (per day) carbohydrates (grams/ day), protein (g/day and % calories), and fat (g/day). Additionally, cholesterol (mg/day), choline (mg/day) and zinc (mg/day) consumption was higher in males than females. There were no significant differences in the consumption of B vitamins, calcium, magnesium, copper, beta-carotene, vitamins C and E, ω-3 fatty acids, fiber, or lutein/zeaxanthin. VCP was significantly better for males than the females and males performed significantly better on the three baseline and three final cognitive performance sessions. However, the change from baseline to the final sessions was similar between males and females.

**Table 2:** Participants average dietary intake and cognitive performance.

		Male (*n* = 38)		Female (*n* = 60)		
		Mean	SD	Mean	SD	P (sex)
Macronutrients	Calories (kcal•day^−1^)	2214	492	1727	402	<0.001
	Calories (kcal•kg^−1^)	28.1	7.0	29.0	7.4	0.554
	Carbs (g•day^−1^)	249.5	75.6	211.5	63.7	0.008
	Carbs (g•kg^−1^•day^−1^)	3.2	1.2	3.6	1.1	0.135
	Carbs (% Calories)	42.3	9.2	45.8	8.4	0.056
	Sugars (g•day^−1^)	77.8	37.7	73.3	29.3	0.507
	Fiber	21.2	8.91	21.4	10.4	0.902
	Protein (g•day^−1^)	122.2	49.1	79.8	27.2	<0.001
	Protein (g•kg^−1^•day^−1^)	1.5	0.6	1.3	0.4	0.036
	Protein (g•kg lean^−1^•day^−1^)	1.8	0.6	1.7	0.6	0.782
	Protein (% Calories)	23.4	8.4	19.3	4.4	0.002
	Fat (g•day^−1^)	81.4	24.0	65.4	22.9	0.001
	Fat (% Calories)	33.5	6.0	34.3	6.6	0.564
Micronutrients	Omega-3 (g•day^−1^)	0.4	0.6	0.3	0.4	0.176
	Cholesterol (mg•day^−1^)	431.0	313.5	232.7	168.8	<0.001
	Choline (mg•day^−1^)	267.7	258.9	142.0	121.4	0.002
	Betaine (mg•day^−1^)	45.9	152.1	20.8	28.2	0.216
	Vitamin B2 (μg •day^−1^)	3.6	10.0	1.4	1.6	0.105
	Vitamin B5 (μg •day^−1^)	5.8	6.8	3.8	5.8	0.136
	Vitamin C (mg•day^−1^)	91.5	85.3	95.6	87.5	0.818
	Vitamin E (mg•day^−1^)	13.1	21.2	11.7	26	0.766
	Copper (mg•day^−1^)	0.9	0.7	0.8	0.6	0.662
	Zinc (mg•day^−1^)	9.3	6.4	5.9	3.1	0.001
	Lut/Zea (μg •day^−1^)	2383	5930	2481	4285	0.925
Cognitive performance	Mean of 15	1.75	0.34	1.52	0.33	0.001
	Maximum of 15	2.38	0.45	2.08	0.43	0.001
	Baseline (3)	1.60	0.40	1.34	0.38	0.001
	Final (3)	1.88	0.35	1.67	0.38	0.007
	Change	0.26	0.30	0.33	0.26	0.185

**Figure 1:**
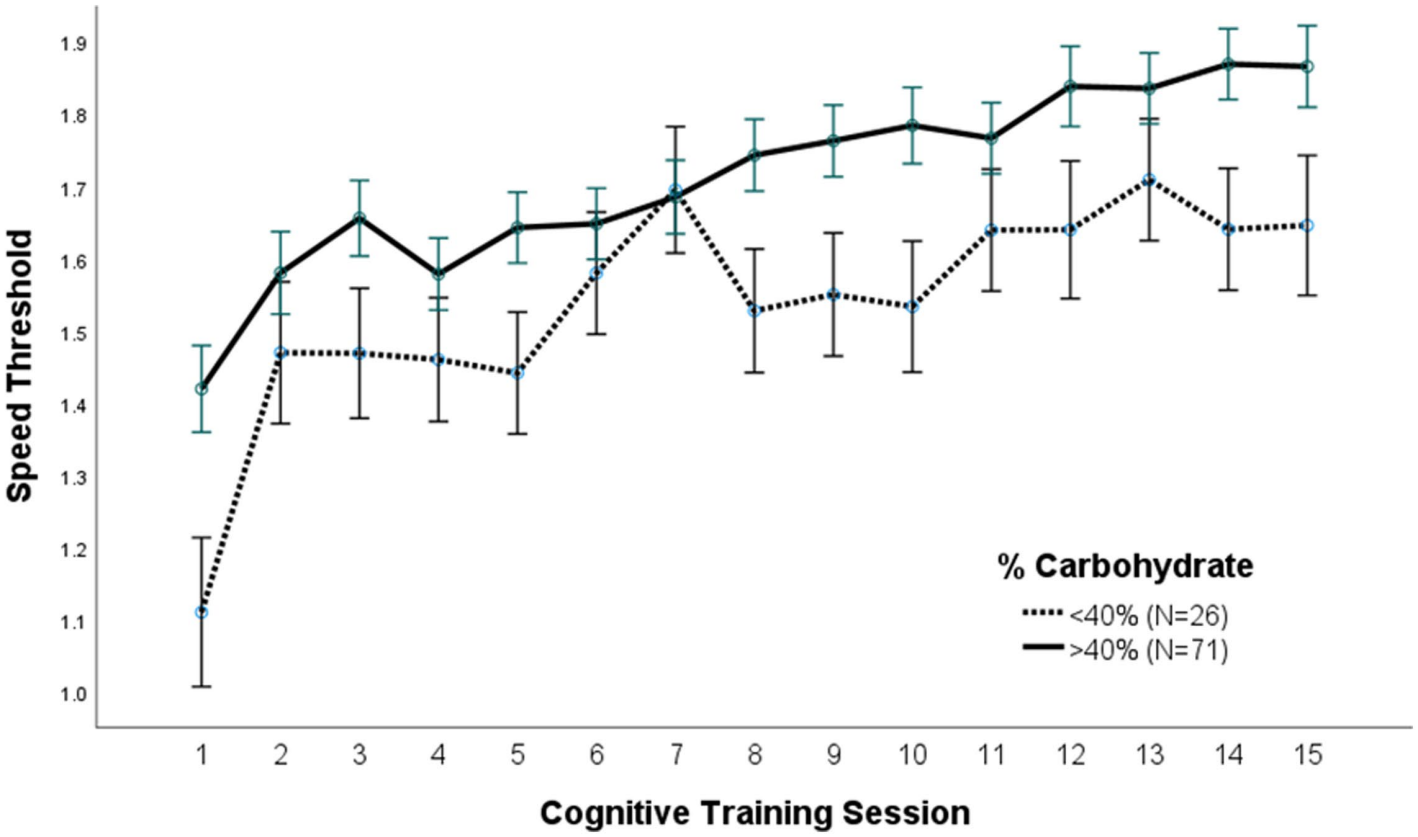
Carbohydrate (% calories) intake. Covariates appearing in the model are evaluated at the following values: Gender = 1.6146, LUTZEA2000 = 1.2708; Error bars: +/− 1 SE. Repeated measures ANOVA *p* = 0.038.

**Figure 2:**
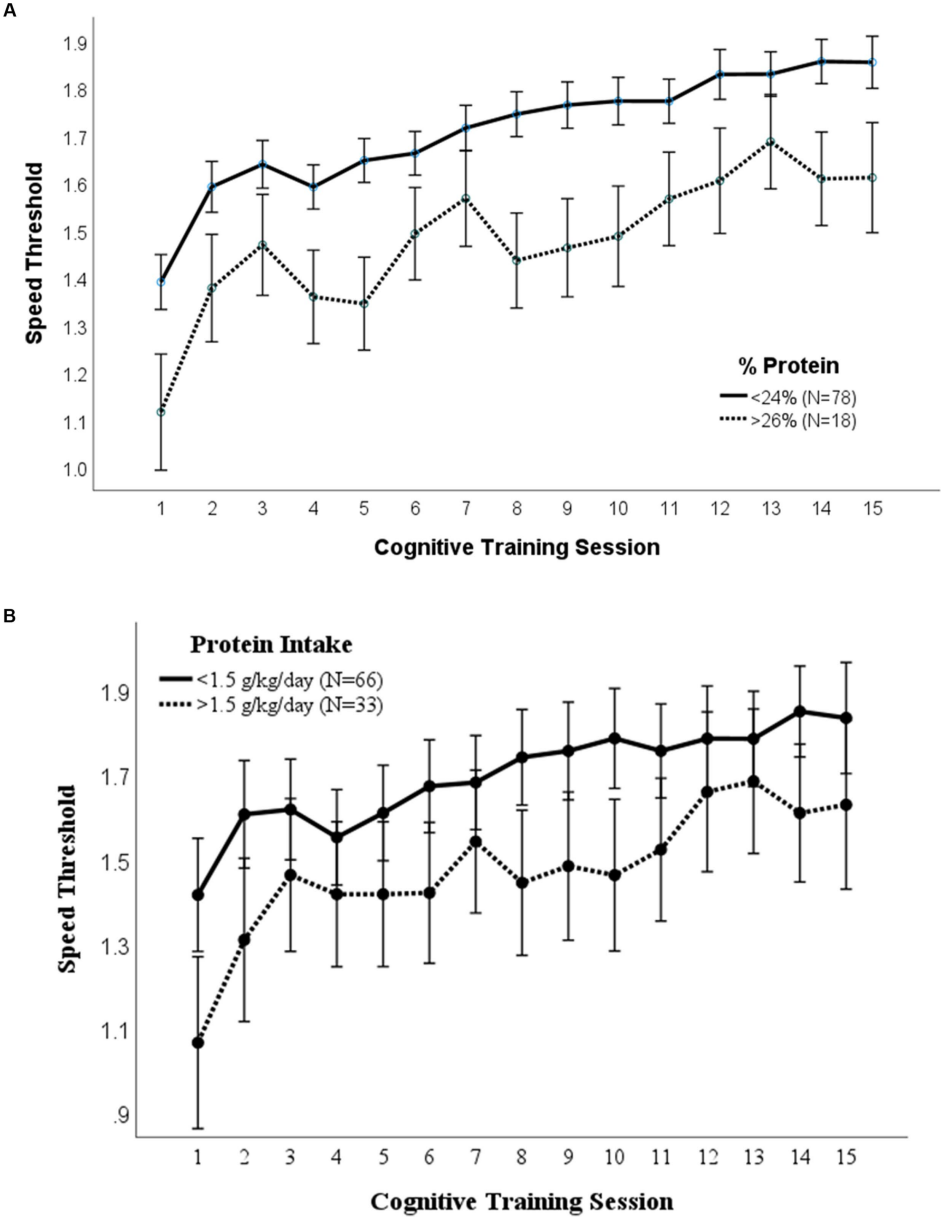
**(A)** Protein (% calories) intake. Covariates appearing in the model are evaluated at the following values: Gender = 1.6146, LUTZEA2000 = 1.2708; Error bars: +/− 1 SE. Repeated measures ANOVA *p* = 0.009. **(B)** Protein (g/kg/day) intake. Covariates appearing in the model are evaluated at the following values: Gender = 1.6146, LUTZEA2000 = 1.2708; Error bars: +/− 1 SE. Repeated measures ANOVA *p* = 0.035.

A repeated measures ANOVA compared the effect of the percent of calories from carbohydrate and protein consumption on visual cognitive performance in Figures 1, 2, respectively. There was a statistically significant difference in cognitive performance and participants who consumed more than 40% carbohydrates (*p* = 0.038) compared to those who consumed less than that amount. A significant breakpoint in the data was identified in those who consumed less than 24% of total calories from protein and those who consumed 26% of total calories from protein. Participants who consumed less than 24% protein performed significantly better on the visual cognitive performance task (*p* = 0.009) than those who consumed 26% calories from protein.

### 3.3. Top performers and cognitive performance

There were also differences in average dietary intake and cognitive performance when comparing the top performers to poor performers. Top performers were defined as the 7 males and 7 females who performed best on 15 cognitive training sessions, while the poor performers were the 8 males and 8 females who performed the worst on the same number of sessions. Mean (*p* < 0.001) and maximal (*p* < 0.001) cognitive performance was significantly higher in top performers when compared to poor performers (Table 3). Top performers also consumed significantly more carbohydrates (% calories) and significantly less protein (g/kg/day and g/kg lean/day) than poor performers (Table 3). There was no significant difference in the consumption of fat, choline, B vitamins, calcium, magnesium, copper, zinc, beta-carotene, vitamins C and E, ω-3 fatty acids, or lutein/zeaxanthin (Table 3).

**Table 3:** Top performers compared to poor performers nutrient intake and cognitive performance.

		Top performers (7 M, &7F)		Poor performers (8 M, 8F)		Covary sex
		Mean	SD	Mean	SD	P (group)
Macronutrients	Calories (kcal•day^−1^)	2087	390	2014	670	0.765
	Calories (kcal•kg^−1^)	30.9	6.3	31.3	8.4	0.896
	Carbs (g•day^−1^)	272	49	231	98	0.174
	Carbs (g•kg^−1^•day^−1^)	4.1	1.1	3.6	1.4	0.337
	Carbs (% Calories)	49.6	6.0	43.0	10.5	0.047*
	Sugars (g•day^−1^)	85.9	30.8	81.3	43.1	0.763
	Protein (g•day^−1^)	88.7	29.6	109	51.8	0.139
	Protein (g•kg^−1^•day^−1^)	1.25	0.33	1.65	0.60	0.035*
	Protein (g•kg lean^−1^•day^−1^)	1.53	0.39	1.99	0.71	0.048*
	Protein (% Calories)	17.8	3.4	23.1	10.7	0.07
	Fat (g•day^−1^)	73.4	17.1	75.6	33.3	0.785
	Fat (% Calories)	32.3	4.2	33.1	7.9	0.762
Micronutrients	Omega-3 (g•day^−1^)	0.30	0.26	0.46	0.74	0.443
	Cholesterol (mg•day^−1^)	240.9	133.7	357.2	322.3	0.158
	Choline (mg •day^−1^)	163	118	208	245	0.493
	Betaine (mg •day^−1^)	85	254	18	17	0.334
	Vitamin B2 (μg •day^−1^)	1.5	1.1	1.6	1.0	0.878
	Vitamin B5 (μg •day^−1^)	3.5	2.3	4.0	2.5	0.547
	Vitamin C (mg •day^−1^)	106.9	124.0	80.7	60.9	0.461
	Vitamin E (mg •day^−1^)	7.2	5.2	9.4	10.1	0.452
	Copper (mg •day^−1^)	0.9	0.7	0.8	0.5	0.401
	Zinc (mg •day^−1^)	7.5	4.5	7.9	5.6	0.779
	Lut/Zea (μg •day^−1^)	2240	2441	1469	1814	0.274
Cognitive performance	Mean of 15	2.22	0.12	1.20	0.17	<0.001*
	Maximum of 15	2.97	0.24	1.72	0.23	<0.001*
	Baseline (3)	2.06	0.28	0.99	0.27	<0.001*
	Final (3)	2.36	0.24	1.31	0.23	<0.001*
	Change	0.30	0.36	0.35	0.13	0.602

*p < 0.05. Top performers were the males and females who performed the best on cognitive testing while poor performers were the males and females who performed the worst on cognitive testing.

### 3.4. Lutein/zeaxanthin intake

**Figure 3:**
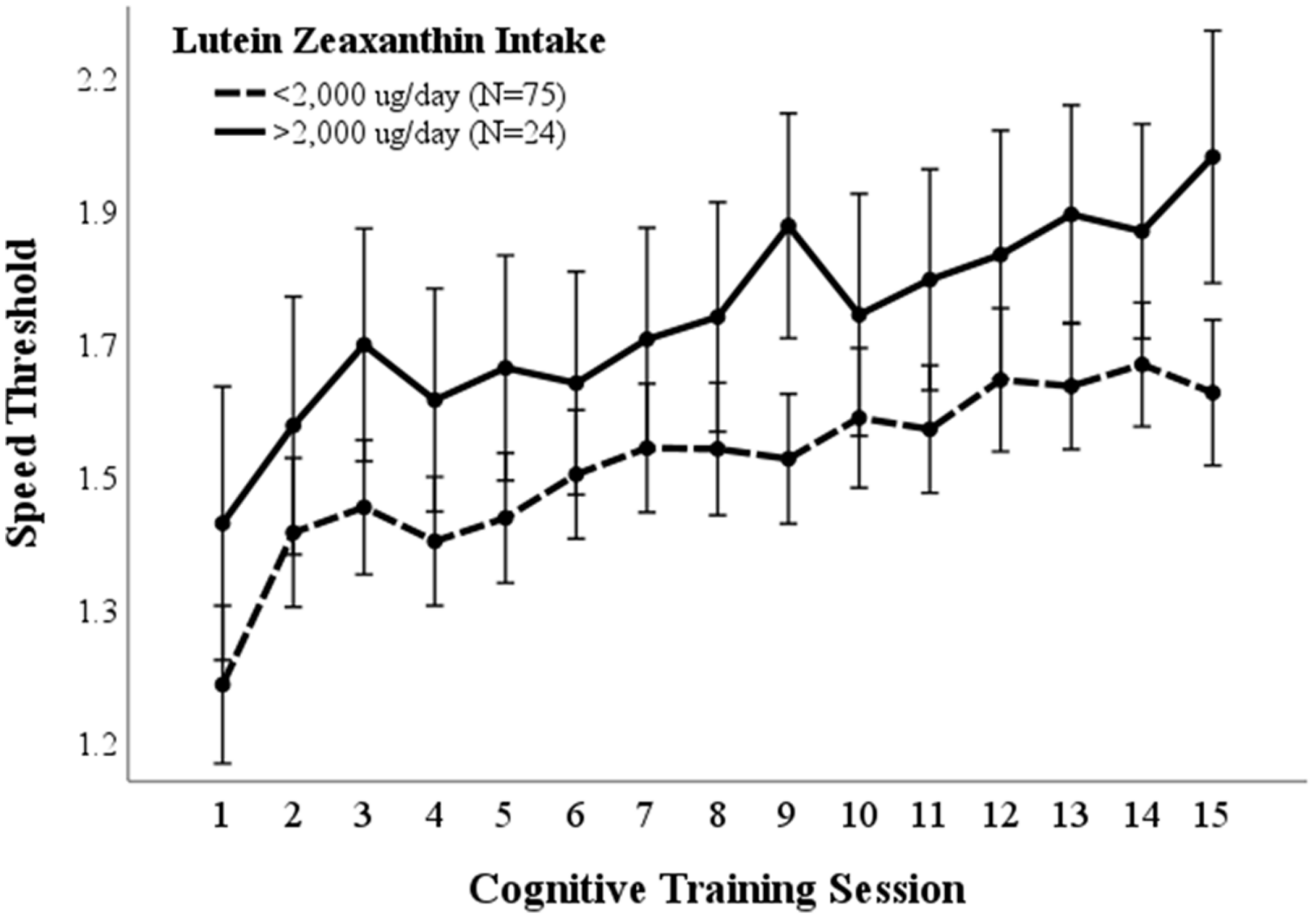
Lutein/Zeaxanthin intake. Covariates appearing in the model are evaluated at the following values: Gender = 1.6146 Error bars: +/− 2 SE. Repeated measures ANOVA *p* = 0.004.

A repeated measures ANOVA compared the effect of lutein/zeaxanthin consumption on visual cognitive performance (Figure 3). Dietary intake of 2,000 μg/day lutein/zeaxanthin was used to distinguish participants who consumed high vs. low lutein/zeaxanthin levels since this is the upper average intake by American adults (34, 35). Mean (*p* = 0.004) and maximal (*p* = 0.031) cognitive performance was significantly higher in those that consumed >2000 μg/day lutein/zeaxanthin. However, both groups improved at the same rate.

### 3.5. Vitamin B2 (riboflavin)

**Figure 4:**
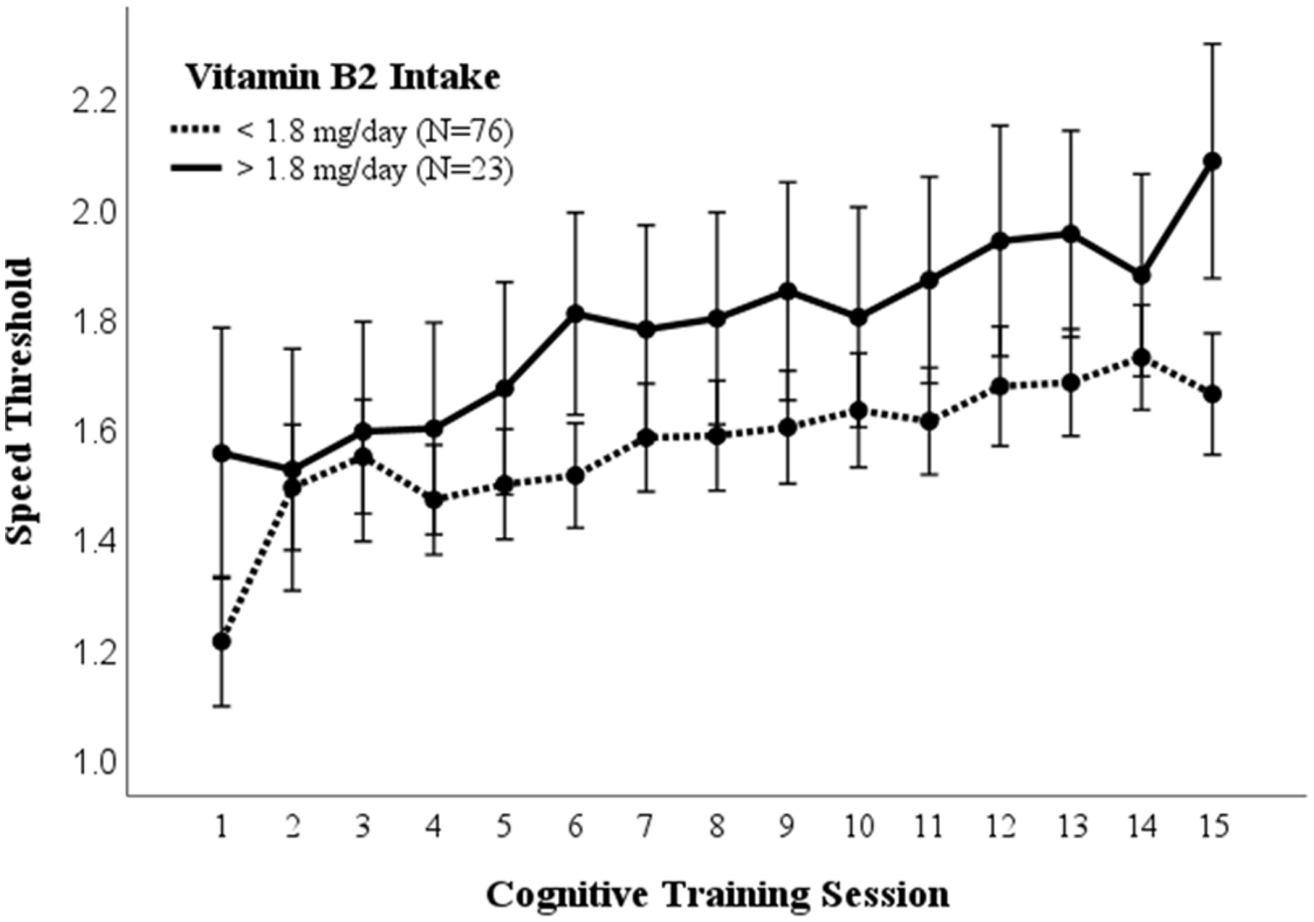
Vitamin B2 intake. Covariates appearing in the model are evaluated at the following values: Gender = 1.6146, PROTEIN = 97.1975 Error bars: +/− 2 SE. Repeated measures ANOVA *p* = 0.014.

A repeated measures ANOVA compared the effect of vitamin B2 (riboflavin) consumption on visual cognitive performance (Figure 4). Dietary intake of 1.8 mg /day was used as a cutoff for high vs. low B2 (riboflavin) consumption based on the average dietary intake for women (36). There was a significantly higher mean (*p* = 0.017) and maximal (*p* = 0.029) cognitive performance in those consuming >1.8 mg/day B2. While the recommended dietary intake for men and women ages 19–51 is 1.3 mg and 1.1 mg, respectively, these differences in cognitive performance were not affected by controlling for sex (EMM ± SE, 0.27 ± 0.03 vs. 0.41 ± 0.06; *p* = 0.032).

### 3.6. Regression

All independent variables that appeared to have an influence on the NT mean and/or maximum speed threshold were considered in the linear regression analysis, only significant/consistent predictors were reported in the linear regression models. There were significant independent effects of sex, protein intake (% kcal), vitamin B2, lutein/zeaxanthin and age on the mean (Table 4) and maximal (Table 5) NT speed threshold; however, lutein/zeaxanthin was not a significant predictor in the model for mean or maximal NT speed thresholds. Although carbohydrate intake positively influenced cognitive performance in the repeated measures ANOVA model, it was not retained in the linear regression models High protein intake was a significant negative predictor of both the mean (Table 4) and the maximal (Table 5) NT speed threshold. Similarly, there was a significant negative relationship between age and the maximal (Table 5) NT speed threshold. Younger subjects had significantly better maximal NT speed threshold than older participants; however, there was not a significant relationship between age and the mean NT speed threshold. Vitamin B2 (riboflavin) had a significantly positive relationship with both the mean (Table 4) and the maximal (Table 5) NT speed threshold.

**Table 4:** Linear regression analysis with neurotracker (NT) mean speed threshold.

Model		Unstandardized coefficients		Standardized coefficients	*t*	Sig.
		B	Std. Error	Beta		
1	(Constant)	2.417	0.326		7.413	<0.001
	Sex (1 = M, 2 = F)	−0.301	0.074	−0.407	−4.073	<0.001
	Protein (% kcal)	−0.019	0.005	−0.346	−3.457	0.001
	Vitamin B2 (1 = low, 2 = high)	0.217	0.089	0.253	2.451	0.017
	Lut/Zea (1 = low, 2 = high)	0.162	0.083	0.189	1.961	0.054
	Age (years)	−0.018	0.011	−0.162	−1.652	0.103

^a^Dependent variable: NT mean.

**Table 5:** Linear regression analysis with neurotracker (NT) maximum speed threshold.

Model		Unstandardized coefficients		Standardized coefficients	*t*	Sig.
		B	Std. Error	Beta		
1	(Constant)	3.523	0.427		8.251	<0.001
	Sex (1 = M, 2 = F)	−0.393	0.097	−0.408	−4.062	<0.001
	Protein (% kcal)	−0.026	0.007	−0.362	−3.600	0.001
	Vitamin B2 (1 = low, 2 = high)	0.317	0.116	0.283	2.728	0.008
	Lut/Zea (1 = low, 2 = high)	0.117	0.108	0.105	1.080	0.284
	Age (years)	−0.030	0.014	−0.215	−2.177	0.033

Dependent variable: NT max.

## 4. Discussion

The results of this study demonstrated a significant association of macronutrients on visual cognitive performance in a group of healthy male and females aged 18–33 who recorded food intake following their normal dietary patterns and completed visual cognitive performance testing on 10 days within a 15-day period. Results of this study demonstrated a significantly positive relationship between individuals who consumed >40% of their macronutrient intake from carbohydrates and visual cognitive performance.

Previous investigations have found that carbohydrates contribute to athletic performance and endurance, but there has been limited emphasis on the relationship between carbohydrate intake and cognitive ability (13). While carbohydrate recommendations for endurance athletes vary based on their intensity and length of performance, recent studies demonstrated a positive relationship between carbohydrate mouth rinses and improved accuracy and precision in cognitive processing (14, 15). However, there is limited research evaluating the relationship of carbohydrate consumption and cognitive performance (15, 16). A small study that evaluated the relationship between three different mouth rinses containing 1.6 g/25 mL carbohydrate, 0.4 g/25 mL guarana complex or 67 mg/25 mL caffeine used at the beginning and twice during a 40-min exercise period reported that all three enhanced temporal activity. The carbohydrate rinse also decreased the rating of perceived exertion (RPE) when compared to the other rinses (16). Another small study that evaluated the impact of maltodextrin mouth rinses also reported that fatigued fencing athletes displayed enhanced accuracy in this skill-based sport after using a maltodextrin mouth rinse (17). Brain imaging of participants using 1.6 g/25 mL maltodextrin mouth rinse displayed enhanced activity in the orbitofrontal cortex of the brain but no influence on reaction rate (18). However, more research on the relationship between habitual carbohydrate consumption and cognitive performance is needed.

Our research showed a significantly negative linear relation between total protein intake and the mean and maximal speed threshold in cognitive testing. Previous investigations reporting a positive relationship between dietary protein consumption and cognitive response were conducted in aging persons, which often consume less protein than the needed protein for maintaining their health (37, 38). However, our population was younger, and many consumed above the RDA for protein, which may have influenced the conflict with the present result. In a study that examined the impact of dietary protein on cognitive performance compared the impact of carbohydrates alone to a protein and carbohydrate mixture, no significant differences in cognitive ability were demonstrated after a 30-min period of exercise (39). Studies evaluating the impact of protein on cognitive performance most often focus on branched chain amino acids (40) whereas we evaluated total protein consumption.

The present results demonstrating better cognitive performance when intake of carbohydrate was >40% and protein <24% is similar to the acceptable macronutrient distribution ranges (AMDR) recommended by the Institute of Medicine including 45–65% carbohydrates, 10–35% protein, and 20–35% fat (40). These percentages also align with other research studies that support the positive relationship of complex carbohydrates with cognitive performance (11, 12). Many diets promote extreme ratios of macronutrients for distinct reasons including, but not limited to, weight loss and athletic performance (14). However, these diets have not evaluated the impact of the varying macronutrient ratios on cognitive performance suggesting additional investigations of macronutrient ratios and cognitive performance is warranted.

Additionally, the results of this study align with other research studies that show a positive relation between lutein/zeaxanthin and enhanced visual cognitive performance (2, 21, 35, 41). For example, a double-blind placebo-controlled study using a lutein and zeaxanthin supplement showed that this daily supplementation for 1 year improved spatial memory, reasoning ability, and complex attention (42). Additionally, a study investigating just 4 months of lutein and zeaxanthin supplementation showed that, compared to placebo, those receiving the intervention significantly increased visual processing speed in healthy young adults (20). Because lutein/zeaxanthin are not endogenously produced (35), individuals must modify their dietary patterns to include lutein/zeaxanthin to receive the benefits of enhanced visual cognitive performance that these nutrients offer. This, in part, explains why much of the literature surrounding cognitive performance and lutein/zeaxanthin utilize supplementation.

Our data suggest a positive relationship between vitamin B2 (riboflavin) and visual cognitive performance. Prior work has demonstrated riboflavin’s role in neurotransmitter synthesis (19). Additionally, dietary riboflavin has been shown to serve as a protective factor for global cognitive ability (43). The relationship between vitamin B2 and cognitive performance resulted in a significant positive result on cognitive performance in those consuming >1.8 mg/day B2, which is greater than the recommended dietary intake for men and women ages 19–51 years. Vitamin B2 is essential for macronutrient metabolism, energy generation, and many other physiological functions including but not limited to antioxidant and anti-inflammatory roles (44).

Limitations of this study include the subject population and the length of the study. The young, college-enrolled population limits the generalization of the results to other age and demographic groups. While we used 10 days of food logs in analyzing dietary intake, reliance on dietary recall to assess nutrient status may result in potential errors due to underreporting which is common in self-reported food records. However, we provided baseline participant education on the level of detail required for reporting and handouts with portion size estimates were given to participants. Food logs were also checked upon submission for detail. Additionally, 10 food records greatly exceed the standard 3 food records for dietary assessment and may capture more of the variability and reflect longer term intake better. Furthermore, this study is limited by the lack of biochemical assessment indicators such as serum analysis and macular pigment optical density for objective evaluation (42, 45–47).

This study provided evidence that several nutrients, particularly lutein/zeaxanthin, vitamin B2, protein, and carbohydrates, contribute to enhanced visual cognitive performance. Athletes who often manipulate macronutrient ratios for muscular performance gains may consider optimizing this balance of protein for muscular gains and carbohydrate for both high energy metabolism and visual cognitive performance. Other micronutrients including lutein/zeaxanthin and B2 offer additional opportunities to enhance/optimize performance of cognitive function outside the traditional skeletal muscle and cardiovascular nutritional interventions and may be highly relevant in aging populations that are impacted by decline in visual cognitive performance.

## Data availability statement

The raw data supporting the conclusions of this article will be made available by the authors, without undue reservation.

## Ethics statement

The studies involving human participants were reviewed and approved by the Texas A&M University Human Subject’s Institutional Review Board. The patients/participants provided their written informed consent to participate in this study.

## Author contributions

NG conducted the research. CS and KB coordinated and supervised the research. KB drafted the manuscript. JG and AR edited the manuscript. SR conception and design of the research, analyzed the data, and interpreted the results of experiments. KB and SR edited and approved final version of manuscript. All authors contributed to the article and approved the submitted version.

## Funding

This study was funded by the Texas A&M University.

## Conflict of interest

The authors declare that the research was conducted in the absence of any commercial or financial relationships that could be construed as a potential conflict of interest.

## Publisher’s note

All claims expressed in this article are solely those of the authors and do not necessarily represent those of their affiliated organizations, or those of the publisher, the editors and the reviewers. Any product that may be evaluated in this article, or claim that may be made by its manufacturer, is not guaranteed or endorsed by the publisher.
